# Differential Regulation of DC Function, Adaptive Immunity, and MyD88 Dependence by Two Squalene Emulsion-Based Vaccine Adjuvants

**DOI:** 10.3390/vaccines12050531

**Published:** 2024-05-13

**Authors:** Jayachandra Reddy Nakkala, Yibo Li, Labone Akter, Xinliang Kang, Xinyuan Chen

**Affiliations:** Biomedical & Pharmaceutical Sciences, College of Pharmacy, University of Rhode Island, 7 Greenhouse Road, Avedisian Hall, Room 480, Kingston, RI 02881, USA; jayachandrareddy.nak@uri.edu (J.R.N.); yibo_li@uri.edu (Y.L.); labone_akter@uri.edu (L.A.); xinliang_kang@uri.edu (X.K.)

**Keywords:** MF59, AS03, AddaVax, AddaS03, squalene, emulsion adjuvant, MyD88, α-tocopherol

## Abstract

MF59 and AS03 are squalene emulsion-based vaccine adjuvants with similar compositions and droplet sizes. Despite their broad use in licensed influenza vaccines, few studies compared their adjuvant effects and action mechanisms side by side. Considering the majority of adjuvants act on dendritic cells (DCs) to achieve their adjuvant effects, this study compared AddaVax and AddaS03 with similar compositions to MF59 and AS03 adjuvants to enhance antigen uptake, DC maturation, ovalbumin (OVA), and seasonal influenza vaccine-induced immune responses. Considering MF59 was reported to activate MyD88 to mediate its adjuvant effects, this study also investigated whether the above-explored adjuvant effects of AddaVax and AddaS03 depended on MyD88. We found AddaVax more potently enhanced antigen uptake at the local injection site, while AddaS03 more potently enhanced antigen uptake in the draining lymph nodes. AddaS03 but not AddaVax stimulated DC maturation. Adjuvant-enhanced antigen uptake was MyD88 independent, while AddaS03-induced DC maturation was MyD88 dependent. AddaVax and AddaS03 similarly enhanced OVA-induced IgG and subtype IgG1 antibody responses as well as influenza vaccine-induced hemagglutination inhibition antibody titers, while AddaS03 more potently enhanced OVA-specific IgG2c antibody responses. Both adjuvants depended on MyD88 to enhance vaccine-induced antibody responses, while AddaVax depended more on MyD88 to achieve its adjuvant effects. Our study reveals similarities and differences of the two squalene emulsion-based vaccine adjuvants, contributing to our improved understanding of their action mechanisms.

## 1. Introduction

Emulsion-based vaccine adjuvants have a long history of use in experimental vaccines since their first development by Freund in the 1930s [1,2]. Complete Freund’s Adjuvant (CFA) is a water-in-oil (w/o) emulsion prepared by emulsifying heat-killed Mycobacterium tuberculosis in non-metabolizable minimal oil (paraffin oil and mannide monooleate) [1,2]. Although CFA vigorously enhances vaccine-induced humoral and cellular immune responses, it persists at the local injection site, induces excessive inflammation, and causes local adverse reactions, such as granulomatous reactions, sterile abscesses, skin ulceration, and pain [1,2]. Incomplete Freund’s Adjuvant (IFA) lacks Mycobacterium components and induces less but still significant local adverse reactions, such as granulomatous reactions and sterile abscesses [1,2]. Other w/o emulsion adjuvants, Montanide ISA 51 and Montanide ISA 720, were developed using a highly purified mineral oil and a metabolizable squalene oil, respectively [3,4]. These adjuvants also show an unsatisfactory safety profile and have been mainly used in cancer vaccines [3,4]. Considering w/o emulsion adjuvants tend to persist at the local injection site and induce adverse reactions [2], o/w emulsion adjuvants were developed for faster local clearance and improved safety. Ribi Adjuvant System (RAS) was developed around 1985 by emulsifying a metabolizable squalene oil and Tween 80 in water and further supplemented with trehalose 6,6′-dimycolate (TDM), monophosphoryl lipid A (MPL), or cell wall skeleton (CWS) to enhance adjuvant potency [5]. RAS also induces significant local adverse reactions, although less severe than CFA [5]. 

These early efforts led to the development of a safe o/w emulsion adjuvant MF59 (Novartis Vaccines and Diagnostics, Basel, Switzerland), which was first approved in 1997 in Europe and later approved in 2020 in the United States to enhance seasonal influenza vaccine efficacy in the elderly [6,7]. MF59 adjuvant has been used in millions of human vaccine doses with good safety profiles [8,9]. A similar o/w emulsion adjuvant AS03 (GlaxoSmithKline Biologicals, Brenttford, UK) was approved to boost the influenza pandemic 2009 H1N1 (pdm09) vaccine efficacy in Europe and approved in 2013 to boost pre-pandemic H5N1 vaccine efficacy in the United States [6,7]. Both MF59 and AS03 are squalene o/w emulsion adjuvants with similar compositions and droplet sizes [9,10]. MF59 contains Tween 80 and Span 85 as surfactants, while AS03 contains Tween 80 and α-tocopherol (vitamin E) [9,11]. Besides potentiation of influenza vaccine-induced immune responses, MF59 and AS03 adjuvants are able to broaden vaccine-induced immune responses against non-vaccine viral strains [7]. Indirect comparison of antigen-dose sparing of pdm09 vaccine favored AS03 over MF59 in six of eight comparisons in adults and favored MF59 over AS03 in two of seven comparisons in pediatrics [12]. Side-by-side comparisons of MF59 and AS03-adjuvanted inactivated monovalent influenza H5N1 and H7N9 vaccines in humans found AS03 was superior to MF59 in induction of more potent and/or broader antibody responses [13,14]. AS03-adjuvanted inactivated monovalent H5N8 vaccine induced higher seroprotection rates than MF59-adjuvanted vaccine in human studies [15]. 

Biodistribution studies found both MF59 and AS03 adjuvants could be rapidly cleared from the local injection site with a clearance half-life of 12.9 h for MF59 and 1.5 h for AS03 [16]. This study indicates AS03 adjuvant can be more quickly cleared from the local injection site despite its subtle difference in composition from MF59. Significant progress has been made in understanding their action mechanisms. Intramuscular (IM) injection of MF59 and AS03 was found to induce strong cytokine/chemokine release and recruit neutrophils, eosinophils, and monocytes to local injection sites and/or draining lymph nodes (LNs) [17,18]. MF59 was found not to activate TLR-mediated signaling pathways and, interestingly, activate MyD88 to mediate its adjuvant effects [19]. MF59 also induced a rapid release of ATP to mediate local cell recruitment and enhance humoral immune responses, despite unknown roles of ATP in MyD88 activation [20]. A recent study further found MF59 could induce receptor-interacting serine-threonine kinase 3 (RIPK3)-dependent necroptosis in the lymph node, which was crucial for cross-presentation of antigen to CD8^+^ T cells [21]. AS03 adjuvant was found to activate endoplasmic reticulum stress sensor inositol requiring enzyme 1α (IRE1α) to mediate its immunostimulatory effects [22].

Due to a similar composition, it remains to be explored whether MF59 and AS03 induce similar mechanisms to mediate their adjuvant effects. Due to a slight difference in composition, it also remains to be explored whether the two adjuvants induce differential mechanisms to mediate their adjuvant effects. Due to the crucial roles of dendritic cells (DCs) in bridging innate and adaptive immunity [23] and the unavailability of MF59 and AS03 adjuvants, this study explored the adjuvant effects of AddaVax and AddaS03 with similar compositions to MF59 and AS03 adjuvants to enhance the antigen uptake, DC maturation, vaccine-induced adaptive immunity, and MyD88 dependence of the diverse adjuvant effects.

## 2. Materials and Methods

### 2.1. Reagents

The trivalent inactivated influenza vaccine (TIV, 2011–2012, NR-36747) was purchased from BEI Resources (Manassas, VA, USA). Influenza A viruses (A/California/07/2009 (H1N1, FR-201), A/Victoria/210/2009 X-187 (H3N2, FR-644)) and influenza B/Brisbane/60/2008 viruses (Victoria Lineage, FR-177) were purchased from International Reagent Resource (IRR, Manassas, VA, USA). Endotoxin-free OVA (vac-pova) was purchased from Invivogen (San Diego, CA, USA). Chicken red blood cells (RBCs, 10100768) were purchased from Charles River Laboratories (Wilmington, MA, USA). Receptor Destroying Enzyme II (RDE II, 370013) was purchased from Hardy Diagnostics (Albany, NY, USA). Fluorescence-conjugated antibodies CD11c (N418), CD40 (3/23), CD80 (16-10A1), CD86 (GL-1), and MHC II (I-A/I-E) (M5/114.15.2) were purchased from Biolegend (San Diego, CA, USA). Alexa Fluor 647 (AF647)-OVA and 1-Step Ultra^TM^ TMB were purchased from Thermo Fisher Scientific (Waltham, MA, USA). Collagenase type 2 (NC9693955) was purchased from Fisher Scientific (Waltham, MA, USA). 

### 2.2. Adjuvants

AddaVax (vac-adx-10) and AddaS03 (vac-as03-10) (both manufactured by Invivogen) were used in our studies due to the unavailability of MF59 and AS03 adjuvants. The composition and nanoemulsion droplet size of AddaVax and AddaS03 and their corresponding commercial products were shown in Table 1. 

### 2.3. Mice

C57BL/6 mice (male, 6–8 weeks old) were purchased from Jackson Laboratory (Bar Harbor, ME, USA). MyD88 knockout (KO) mice were purchased from Jackson Laboratory (Bar Harbor, ME, USA) and self-bred for use in this study. Anesthesia was induced by intraperitoneal injection of a mixture of ketamine (80 mg/kg) and xylazine (10 mg/kg). Influenza viral challenge studies were performed in the animal biosafety level 2 (ABSL-2) facility of the University of Rhode Island (URI). All animal procedures were approved by the Institutional Animal Care and Use Committee (IACUC) of URI (AN1415-009) and conducted in accordance with national and institutional guidelines and regulations.

### 2.4. Immunization

C57BL/6 (WT) and MyD88 KO mice (male, 6–8 weeks old) were intramuscularly immunized with OVA (10 µg) or TIV of 2011–2012 season (0.3 μg hemagglutinin (HA) per strain) alone or admixed with AddaVax or AddaS03 adjuvant. TIV comprised viral antigens from influenza A/California/07/2009 X-179A H1N1 (pdm09), A/Victoria/210/2009 X-187 H3N2 (A/Perth/16/2009-like virus), and B/Brisbane/60/2008 viruses (Victoria lineage). Adjuvants were mixed with TIV at 1:1 volume ratio right before immunization.

### 2.5. Serum Antibody Titer

A small volume of blood (~50 μL) was collected for measurement of serum antibody titer following procedures described in our previous reports [24,25]. In brief, 96-well plates were coated with 100 μg/mL OVA followed by blocking and incubation with 4-fold serial diluted serum samples. Plates were next incubated with horseradish peroxidase (HRP)-conjugated anti-mouse IgG, IgG1, and IgG2c secondary antibodies. Plates were then incubated with TMB substrates. Reactions were stopped by adding 3N H_2_SO_4_ followed by reading OD_450nm_ in a microplate reader (Molecular Device, San Jose, CA, USA). 

### 2.6. Hemagglutination Inhibition (HAI) Titer

Serum HAI titers were measured as in our previous reports [26,27]. Briefly, serum samples were incubated with RDE II followed by heat inactivation. Serum samples were then adsorbed with chicken RBCs to remove non-specific binding. Serum samples were then subjected to two-fold serial dilutions followed by incubation with 4 hemagglutination units of influenza A/H1N1, A/H3N2, and B/Victoria viruses. Chicken RBCs were added and HAI titers were determined as the reciprocal of the highest dilution that completely inhibited agglutination of chicken RBCs. 

### 2.7. Lethal Viral Challenge

Influenza viral challenge was performed as in our previous reports [25,28]. Briefly, mice were anesthetized and inoculated intranasally with 10× LD_50_ of mouse-adapted pdm09 viruses. Body weight and survival were monitored daily for 14 days. Mice were euthanized and considered dead if their body-weight loss was more than 25%.

### 2.8. Single-Cell Suspension Preparation

WT and MyD88 KO mice were intramuscularly injected with 2 μg AF647-OVA alone or in the presence of AddaVax or AddaS03 adjuvant. Quadriceps muscle was collected 15 h after immunization and single-cell suspension was prepared following a published protocol [29]. Briefly, quadriceps muscle was cut into small pieces along the skeletal muscle fiber and digested in 5 mL muscle dissociation buffer (Hanks’ Balanced Salt Solution (HBSS) supplemented with 1% penicillin/streptomycin, 10% Fetal Bovine Serum (FBS), and 725 U/mL Collagenase type 2) at 37 °C for 1 h with constant shaking (100 rpm). Digestion was stopped by adding 10 mL cold HBSS supplemented with 1% penicillin/streptomycin and 10% FBS. After centrifugation at 525× *g* for 5 min at room temperature, 11 mL supernatants were discarded and 0.5 mL Collagenase type 2 (100 U/mL) and 0.5 mL Dispase (1.1 U/mL, Invitrogen, Carlsbad, CA, USA) were added. The pellets were gently suspended and further digested at 37 °C for 30 min with constant shaking (100 rpm). Samples were collected into a 10 mL syringe and passed through an 18-gauge needle 10–12 times to break any remaining muscle. Then the reaction was stopped by adding 10 mL cold HBSS supplemented with 1% penicillin/streptomycin and 10% FBS. After centrifugation at 525× *g* for 5 min at room temperature, 11 mL supernatants were removed and the remaining 4 mL medium was gently mixed. The samples were then passed through 70 μm cell strainer. Cells were washed with FACS buffer (phosphate-buffered saline (PBS) supplemented with 2% FBS) followed by immunostaining. Popliteal and inguinal draining LNs were collected at the same time of muscle collection. LNs were pressed through 70 μm cell strainer to prepare single-cell suspensions as in our previous reports [26,27]. 

### 2.9. Immunostaining and Flow Cytometry

Immunostaining and flow cytometry were performed similarly as in our previous reports [27,30]. Briefly, single-cell suspensions of muscle and LNs were stained with fluorescence-conjugated anti-CD11c, CD40, CD80, CD86, and MHC II antibodies followed by fixation in 2% formaldehyde. Cells were then subjected to flow cytometry analysis in BD FACSVerse. FlowJo software v10 was used to analyze the data. 

### 2.10. Statistical Analysis

Values were expressed as Mean ± SEM (standard error of the mean). Two-way ANOVA with Tukey’s multiple comparison test was used to compare differences between groups or otherwise specified. *p* value was calculated by PRISM software (GraphPad, San Diego, CA, USA) and considered significant when it was less than 0.05.

## 3. Results

### 3.1. AddaVax and AddaS03 Differentially Increase Antigen Uptake and DC Maturation at Injection Site

The majority of vaccine adjuvants act on DCs to improve antigen uptake, maturation, and migration [23,31,32]. Here, we first explored a potential impact of AddaVax and AddaS03 on local antigen uptake following IM injection of AF647−OVA alone or in the presence of either adjuvant. Quadriceps muscle was collected 15 h after injection followed by single-cell suspension preparation, immunostaining, and flow cytometry analysis. Commonly used markers CD11c and MHC II were used to identify pan−DCs (CD11c^+^ MHC II^+^) [33,34,35]. We found AddaVax significantly increased frequencies of muscle CD11c^+^ MHC II^+^ DCs to a similar extent in WT and MyD88 KO mice (Figure S1), suggesting MyD88 was not crucial for this process. AddaS03 significantly increased muscle DCs in MyD88 KO but not in WT mice (Figure S1), indicating MyD88 might suppress this process.

**Figure 1:**
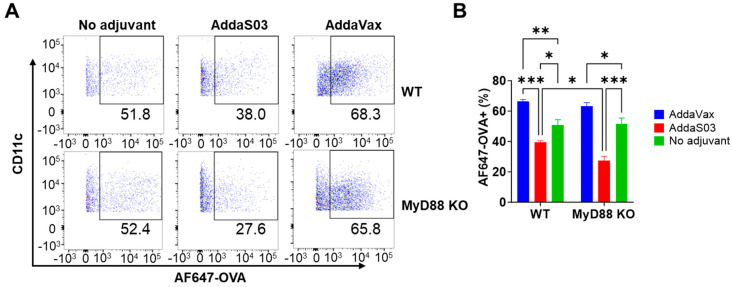
AddaVax increases local antigen uptake in WT and MyD88 KO mice. WT and MyD88 KO mice were intramuscularly injected with AF647−OVA alone or in the presence of AddaVax or AddaS03. Muscle was collected 15 h later, followed by single-cell preparation, immunostaining, and flow cytometry analysis. Cells were first gated based on FSC and SSC and then CD11c and MHC II expression. CD11c^+^ MHC II^+^ cells were gated to analyze percentage of AF647−OVA^+^ cells. (**A**) Representative dot plots of AF647−OVA^+^ cells in CD11c^+^ MHC II^+^ cells in WT (**top**) and MyD88 KO mice (**bottom**). (**B**) Comparison of percentage of AF647−OVA^+^ cells between groups. Two−way ANOVA with Tukey’s multiple comparison test was used to compare differences between groups. n = 6. *, *p* < 0.05; **, *p* < 0.01; ***, *p* < 0.001.

We further found AddaVax similarly increased frequencies of AF647−OVA^+^ DCs in WT and MyD88 KO mice (Figure 1A,B), which is indicative of MyD88 independence. Interestingly, AddaS03 significantly reduced frequencies of AF647−OVA^+^ DCs in WT and MyD88 KO mice with more significant reduction in MyD88 KO mice (Figure 1A,B). These results suggest that MyD88 likely contributes to antigen uptake in the presence of AddaS03 adjuvant, although the underlying mechanisms remain elusive.

**Figure 2:**
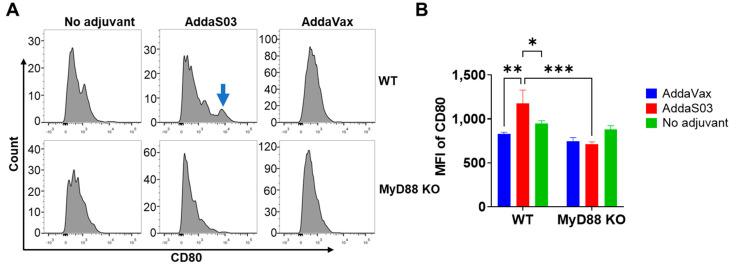
AddaS03 increases CD80 expression on muscle DCs. Muscle DCs were analyzed for surface expression of CD80. (**A**) Representative histograms of CD80 expression on muscle DCs in WT (**top**) and MyD88 KO mice (**bottom**). Arrow: Induced peak of CD80 expression. (**B**) Comparison of MFI of CD80 on muscle DCs between groups. Two−way ANOVA with Tukey’s multiple comparison test was used to compare differences between groups. n = 6. *, *p* < 0.05; **, *p* < 0.01; ***, *p* < 0.001.

AddaS03 was found to significantly increase CD80 expression on muscle DCs in WT but not MyD88 KO mice (Figure 2A,B), which is indicative of MyD88 dependence. We also compared percentages of CD80^hi^ DCs and found similar trends to MFI of CD80 (Figure S2A,B). We further found higher percentages of CD80^hi^ cells in AF647−OVA^+^ than AF647−OVA^−^ DCs (8.85% vs. 2.12%, Figure S2C,D). For other co-stimulatory molecules, AddaVax and AddaS03 slightly reduced CD40 and CD86 expression in muscle DCs with no significant difference between WT and MyD88 KO mice (Figure S3). 

### 3.2. AddaS03 More Potently Increases Antigen Uptake in Draining LNs

**Figure 3:**
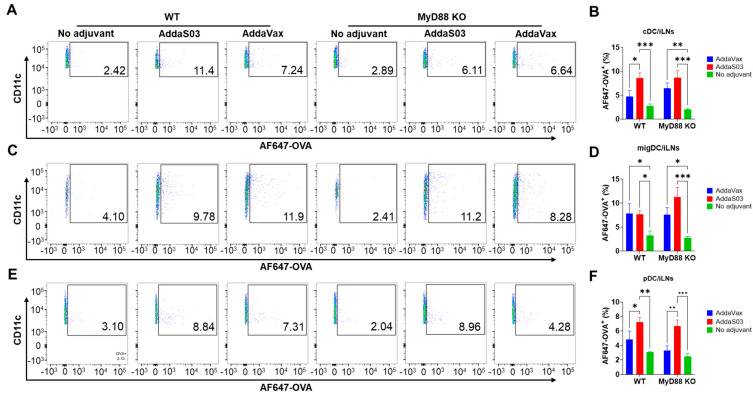
AddaS03 and AddaVax enhance antigen uptake in inguinal LNs. Inguinal draining LNs were collected 15 h after intramuscular injection of AF647−OVA alone or in the presence of AddaVax or AddaS03. Single-cell suspensions were prepared, followed by immunostaining and flow cytometry analysis. Live cells were first gated based on FSC and SSC. cDCs, migDCs, and pDCs were then gated based on CD11c and MHC II expression. Percentages of AF647−OVA^+^ cells in cDCs, migDCs, and pDCs were then analyzed. Representative dot plots of AF647−OVA^+^ cells in cDCs, migDCs, and pDCs are shown in (**A**,**C**,**E**), respectively. Comparisons of percentages of AF647−OVA^+^ cells in cDCs, migDCs, and pDCs are shown in (**B**,**D**,**F**), respectively. Two-way ANOVA with Tukey’s multiple comparison test was used to compare differences between groups. n = 6. *, *p* < 0.05; **, *p* < 0.01; ***, *p* < 0.001.

Mouse quadriceps muscle injection can be drained to popliteal and inguinal LNs [36]. To explore the potential impact of adjuvants on antigen uptake in draining LNs, both LNs were collected 15 h after injection followed by single-cell suspension preparation, immunostaining, and flow cytometry analysis. DCs were divided into three subsets (conventional DC/cDC, migratory DC/migDC, and plasmacytoid DC/pDC) based on their relative CD11c and MHC II expression, as in our previous reports [26,27]. As shown in Figure 3A,B, significantly higher frequencies of AF647−OVA^+^ cDCs in inguinal LNs were found in AddaS03 group than in the no adjuvant group in WT and MyD88 KO mice. AddaVax also increased frequencies of AF647−OVA^+^ cDCs (Figure 3A,B). However, a statistically significant difference was only found in MyD88 KO mice (Figure 3B). Both AddaVax and AddaS03 significantly increased frequencies of AF647−OVA^+^ migDCs in inguinal LNs in WT and MyD88 KO mice (Figure 3C,D). AddaS03 also significantly increased frequencies of AF647−OVA^+^ pDCs in WT and MyD88 KO mice (Figure 3E,F). AddaVax increased frequencies of AF647−OVA^+^ pDCs in WT and MyD88 KO mice (Figure 3F), but the difference did not reach statistically significant level. No significant differences in frequencies of AF647−OVA^+^ cDCs, migDCs, or pDCs were found in no adjuvant, AddaVax, or AddaS03 groups between WT and MyD88 KO mice, suggesting MyD88 independence for enhanced antigen uptake in inguinal LNs. More significant antigen uptake in the presence of AddaS03 was also found in popliteal LNs (Figure S4). AddaS03 significantly increased frequencies of AF647−OVA^+^ cDCs and pDCs in popliteal LNs in WT mice, while AddaVax failed to significantly increase AF647−OVA^+^ cDCs, migDCs, or pDCs in WT or MyD88 KO mice (Figure S4). 

### 3.3. AddaS03 but Not AddaVax Stimulates DC Maturation in Draining LNs

**Figure 4:**
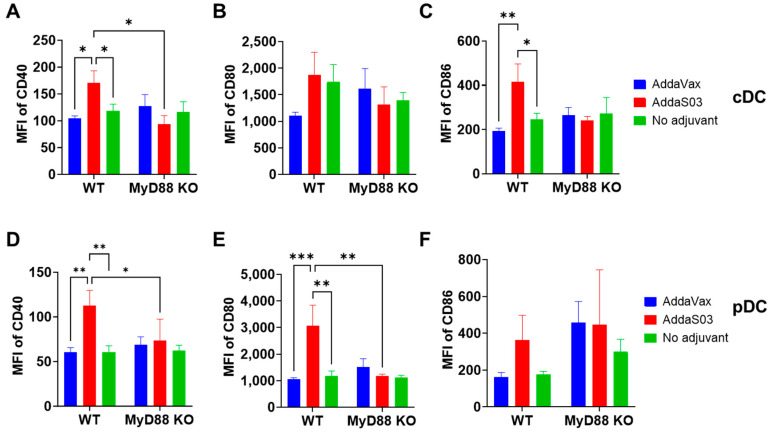
AddaS03 increases co-stimulatory molecule levels in cDCs and pDCs of popliteal LNs. Lymphocytes of popliteal LNs were stained with fluorescence-conjugated antibodies followed by flow cytometry analysis. Cells were first gated based on FSC and SSC. cDCs, migDCs, and pDCs were then gated based on CD11c and MHC II expression. MFI of CD40, CD80, and CD86 in cDCs and pDCs was obtained. MFI of CD40, CD80, and CD86 in cDCs is shown in (**A**–**C**), respectively. MFI of CD40, CD80, and CD86 in pDCs is shown in (**D**–**F**), respectively. Two-way ANOVA with Tukey’s multiple comparison test was used to compare differences between groups. n = 6. *, *p* < 0.05; **, *p* < 0.01; ***, *p* < 0.001.

The impact of adjuvants on DC maturation in popliteal LNs was first analyzed. We found AddaS03 could significantly increase CD40 and CD86 levels in cDCs (Figure 4A–C) and CD40 and CD80 levels on pDCs (Figure 4D–F). The significant enhancement of co-stimulatory molecule expression by AddaS03 only occurred in WT mice (Figure 4A–F), suggesting crucial roles of MyD88 in this process. Interestingly, AddaS03 failed to significantly increase CD40, CD80, or CD86 levels in migDCs (Figure S5), though we found AddaS03 significantly enhanced CD80 expression in muscle DCs (Figure 2). AddaVax failed to significantly increase CD40, CD80, or CD86 levels in cDCs, migDCs, or pDCs in WT or MyD88 KO mice (Figure 4A and Figure S5). 

**Figure 5:**
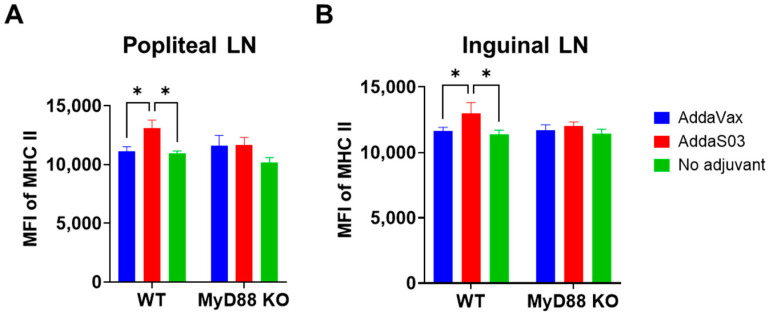
AddaS03 increases MHC II levels in cDCs of draining LNs. Lymphocytes of popliteal and inguinal draining LNs were stained with fluorescence-conjugated antibodies followed by flow cytometry analysis. Cells were first gated based on FSC and SSC. cDCs, migDCs, and pDCs were then gated based on CD11c and MHC II expression. MFI of MHC II in cDCs of popliteal LNs and inguinal LNs was shown in (**A**,**B**), respectively. Two-way ANOVA with Tukey’s multiple comparison test was used to compare difference between groups. n = 6. *, *p* < 0.05.

AddaS03 and AddaVax did not significantly increase co-stimulatory molecule levels in inguinal LNs (Figure S6). Interestingly, we found AddaS03 significantly increased MHC II levels in cDCs but not migDCs or pDCs in popliteal and inguinal LNs (Figure 5A,B). Similarly, the enhanced expression only occurred in WT but not MyD88 KO mice (Figure 5A,B), indicating crucial roles of MyD88 in AddaS03-induced MHC II expression. MFI of MHC II in migDCs or pDCs in popliteal or inguinal LNs showed no significant difference between groups (Figure S7). 

### 3.4. AddaVax More Depends on MyD88 to Enhance OVA-Induced Antibody Responses

**Figure 6:**
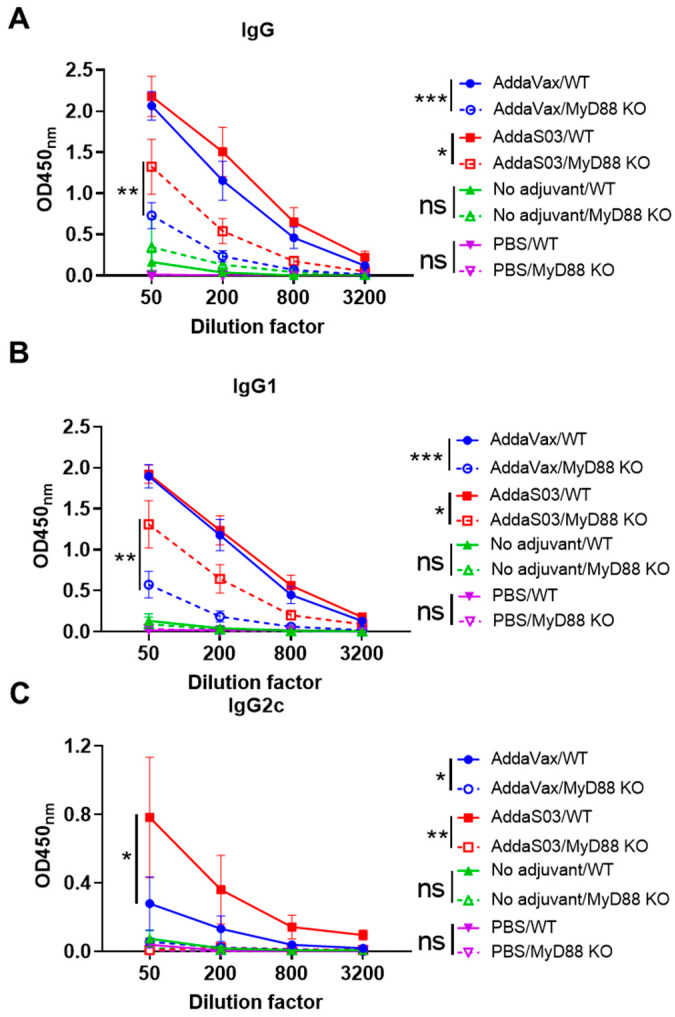
AddaVax depends more on MyD88 to enhance OVA-induced antibody responses. WT and MyD88 KO mice were intramuscularly immunized with 10 µg OVA alone or in the presence of AddaS03 or AddaVax adjuvant or immunized with PBS to serve as a negative control. Serum anti-OVA IgG and subtype IgG1 and IgG2c antibody titer was measured 3 weeks later and shown in (**A**–**C**), respectively. Two-way ANOVA with Bonferroni’s post-test was used to compare differences between groups. n = 5–7. *, *p* < 0.05; **, *p* < 0.01; ***, *p* < 0.001. ns: not significant.

The above studies found both AddaVax and AddaS03 adjuvants enhanced antigen uptake in draining LNs, which was not MyD88 dependent; AddaS03 but not AddaVax increased co-stimulatory molecule levels both locally and in draining LNs, which was highly dependent on MyD88; AddaVax but not AddaS03 significantly increased antigen uptake at local injection site, which was not MyD88 dependent. Next, we compared adjuvant effects of AddaVax and AddaS03 to enhance model antigen OVA-induced antibody responses and their dependence on MyD88. AddaVax and AddaS03 enhanced anti-OVA IgG and IgG1 antibody production to similar levels in WT mice (Figure 6A,B). AddaVax and AddaS03 also enhanced anti-OVA IgG and IgG1 antibody production in MyD88 KO mice (Figure 6A,B), but the enhancement was much weaker than that in WT mice. These results indicated MyD88 played an important role in AddaVax and AddaS03-potentiated anti-OVA IgG and IgG1 antibody production. More significant reduction of anti-OVA IgG and IgG1 antibody levels in MyD88 KO mice in AddaVax than AddaS03 group suggested that AddaVax depended more on MyD88 to enhance OVA-induced IgG and IgG1 antibody production. AddaS03 induced more potent anti-OVA IgG2c antibody production than AddaVax in WT mice (Figure 6C). Anti-OVA IgG2c antibody titer reduced to similar baseline levels in MyD88 KO mice in both adjuvant groups (Figure 6C), suggesting crucial roles of MyD88 for potentiation of OVA-specific IgG2c antibody responses in the presence of AddaVax or AddaS03.

### 3.5. AddaVax Depends More on MyD88 to Enhance Influenza Vaccine-Induced Antibody Responses and Protection

Next, a real vaccine was used to compare the two adjuvant effects and their relative dependence on MyD88. To this end, WT and MyD88 KO mice were intramuscularly immunized with TIV of 2011–2012 flu season alone or in the presence of AddaVax or AddaS03. Serum HAI titers were measured 3 weeks after immunization. 

**Figure 7:**
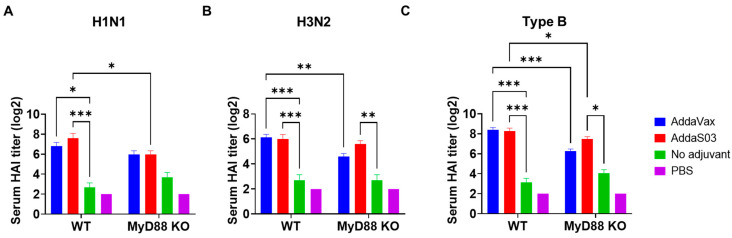
AddaVax depends more on MyD88 to enhance TIV-induced HAI titers. WT and MyD88 KO mice were subjected to IM immunization of TIV (0.3 µg HA per strain) alone or in the presence of AddaVax or AddaS03 adjuvant, or PBS. Serum HAI titer against H1N1, H3N2, type B strain was measured 3 weeks later and shown in (**A**–**C**), respectively. Two-way ANOVA with Fisher’s LSD test was used to compare differences between groups. n = 5–7. *, *p* < 0.05; **, *p* < 0.01; ***, *p* < 0.001.

AddaVax and AddaS03 similarly enhanced HAI titers against H1N1, H3N2, and type B strains in WT mice (Figure 7A–C). Significantly reduced HAI titers against H3N2 and type B strains were found in MyD88 KO as compared to WT mice in AddaVax groups (Figure 7B,C). Significantly reduced HAI titers against H1N1 and type B strains were found in MyD88 KO as compared to WT mice in AddaS03 groups (Figure 7A,C). These results indicated MyD88 played an important role in AddaVax and AddaS03 adjuvant effects to enhance TIV-induced antibody responses. AddaS03 significantly increased serum HAI titer against H3N2 and type B strains in MyD88 KO mice (Figure 7B,C), while AddaVax failed to significantly increase serum HAI titer against all strains in MyD88 KO mice (Figure 7A–C). This discrepancy suggested that AddaVax depended more on MyD88 to enhance TIV-induced antibody responses.

**Figure 8:**
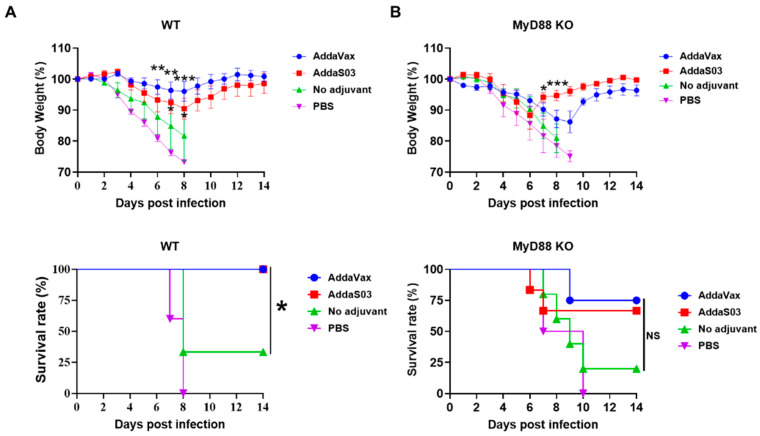
AddaVax more depends on MyD88 to enhance TIV-induced protection against pdm09 viral challenges. WT and MyD88 KO mice (the same mice in Figure 7) were challenged with a lethal dose (10× LD_50_) of mouse-adapted pdm09 viruses. Body weight change (**top**) and survival (**bottom**) were monitored daily for 14 days in WT (**A**) and MyD88 KO mice (**B**); n = 5–7. Two-way ANOVA with Fisher’s LSD test was used to compare differences in body weight loss on individual days between groups. Log-rank test with Bonferroni’s correction was used to compare differences of survival between no adjuvant and adjuvant groups. *, *p* < 0.05; **, *p* < 0.01; ***, *p* < 0.001. NS: not significant.

The immunized WT and MyD88 KO mice were then challenged with a lethal dose of pdm09 viruses 4 weeks after immunization. TIV immunization in the presence of AddaVax or AddaS03 showed significantly reduced body weight loss compared with TIV immunization alone in WT mice (Figure 8A). Significantly reduced body weight loss was observed 6–8 days post infection (dpi) in the AddaVax group and 7 and 8 dpi in the AddaS03 group when compared to the no adjuvant group (Figure 8A). No significant difference in body weight loss was found between the two adjuvant groups, though mice in the AddaVax group lost a maximal 4% body weight and mice in the AddaS03 group lost a maximal 10% body weight (top, Figure 8A). All mice in the AddaVax or AddaS03 group survived the lethal viral challenges, while only 33% mice survived the lethal viral challenges in the no adjuvant group (bottom, Figure 8A). Significantly reduced protection was observed in MyD88 mice in adjuvant groups. MyD88 KO mice in the AddaVax group lost a maximal 14% of body weight and those in the AddaS03 group lost a maximal 12% body weight (top, Figure 8B). Significantly reduced body weight loss of MyD88 KO mice was only observed in the AddaS03 group when compared to the no adjuvant group (top, Figure 8B). Furthermore, 75% of MyD88 KO mice in the AddaVax group survived the lethal viral challenge and 67% of MyD88 KO mice in the AddaS03 group survived the lethal viral challenge, while only 20% of MyD88 KO mice in the no adjuvant group survived the challenge (bottom, Figure 8B). The overall more significant reduction of protection in MyD88 KO mice in AddaVax than AddaS03 group suggested that AddaVax adjuvant depended more on MyD88 to enhance TIV-induced protection against pdm09 viral challenges. 

## 4. Discussion

Side-by-side comparison of AddaVax and AddaS03 adjuvants reveals their similarities and differences in potentiation of DC function and adaptive immunity. Our major findings are that the AddaVax adjuvant more significantly enhanced local antigen uptake, while the AddaS03 adjuvant more significantly enhanced antigen uptake in draining LNs; AddaS03 induced DC maturation at local injection site and in draining LNs, while AddaVax failed to induce DC maturation; AddaS03-induced DC maturation was highly dependent on MyD88; AddaVax and AddaS03-potentiated adaptive immune responses depended on MyD88; and AddaVax depended more on MyD88 to enhance vaccine-induced immune responses.

AddaVax vigorously enhanced local antigen uptake 15 h after injection, while AddaS03 showed a weak opposite effect. This might be related to their differential local clearance rate. AS03 can be more rapidly cleared with a half-life of only 1.5 h, while MF59 has a clearance half-life of 12.9 h [16]. Due to the similar composition and droplet sizes, AddaVax and AddaS03 likely have similar local clearance rates to their respective licensed products. Longer deposition allowed AddaVax to have a durable effect on local DCs, which might be crucial for the enhanced antigen uptake. Besides enhancing antigen uptake, AddaVax also increased local DC levels by more than 2-fold at 15 h, while AddaS03 showed a minimal effect. A significant increase of local DC levels by AddaVax was in line with prior reports that MF59 could recruit monocytes to differentiate into DCs [9,17]. The lack of a significant increase in local DC levels by AddaS03 may reflect its quick clearance from the local injection site. Despite its quick clearance, AddaS03 significantly increased CD80 expression in DCs at the local injection site. This indicates the initial transient exposure of AddaS03 is sufficient to increase CD80 expression but not antigen uptake. Antigen uptake in the draining LNs showed a different trend from local antigen uptake. AddaS03 similarly or more potently enhanced antigen uptake in different DC subsets in popliteal and inguinal draining LNs. MyD88 showed a dispensable role in adjuvant-enhanced antigen uptake at the local injection site or in draining LNs. 

AddaS03 significantly increased co-stimulatory molecule expression of DCs in the draining LNs, while AddaVax showed a minimal effect. The ability of AddaS03 to stimulate co-stimulatory molecule expression (i.e., DC maturation) is highly likely due to the effects of α-tocopherol, considering the other two components (squalene and Tween 80) are also contained in AddaVax. α-tocopherol is the most bioavailable vitamin E and has complex interplays with immune systems [37,38]. α-tocopherol was found to stimulate DC maturation at low concentrations in vitro [39]. A prior study found α-tocopherol in AS03 could activate monocytes but not DCs to secrete cytokine/chemokines and increase antigen uptake in monocytes but not DCs [18]. A recent study found α-tocopherol in a squalene emulsion adjuvant drove the induction of T follicular helper (Tfh) cells and germinal center B (GCB) cells and also contributed to the recruitment of neutrophils, eosinophils, and monocytes to draining LNs [40]. To our knowledge, this is the first time that α-tocopherol-containing squalene emulsion adjuvant (AddaS03) was found to stimulate DC maturation in vivo. Furthermore, AddaS03-stimulated DC maturation was highly dependent on MyD88. Considering AddaVax failed to stimulate DC maturation, we believe α-tocopherol activates MyD88-dependent signaling pathways to mediate DC maturation. 

OVA and influenza vaccination studies found MyD88 played an important role in AddaVax and AddaS03 adjuvant effects. A lack of MyD88 significantly reduced OVA and TIV-induced antibody responses in the AddaVax or AddaS03 group. Due to a crucial role of MyD88 in MF59 adjuvant effects, it is highly plausible that MyD88 also played a crucial role in AS03 adjuvant effects. To our knowledge, this is the first study to prove that MyD88 also plays a crucial role in AddaS03 adjuvant effects. Interestingly, AddaVax seems more dependent on Myd88 to enhance OVA and TIV-induced antibody responses and TIV-induced protection than AddaS03. This result suggests that AddaS03 likely stimulates MyD88-indepdent mechanisms to mediate its adjuvant effects. This is likely mediated by α-tocopherol. 

Regarding the relative adjuvant potency, AddaS03 was found to enhance OVA-specific IgG2c antibody responses more potently than AddaVax. AddaS03 also induced higher anti-OVA IgG and HAI titer against H1N1 strain than AddaVax, but the differences did not reach statistically significant levels. Overall, AddaS03 and AddaVax showed similar adjuvant effects, enhancing OVA and TIV-induced antibody responses. Similar adjuvant effects seemed to contrast with the overall more potent antigen uptake and DC maturation in the draining LNs of AddaS03 group. One plausible explanation is AddaVax adjuvant may induce more efficient antigen processing and presentation. Prior studies found both MF59 and AS03 adjuvants could expand avian influenza H5N1 vaccine-induced antibody repertoires to target HA1 sites after screening whole-genome-fragment phage display libraries to identify the binding regions of elicited antibodies [41,42]. Yet, an indirect comparison found MF59 induced a higher frequency of HA1-to-HA2-specific phage clones than AS03 when compared with the no adjuvant group (12- vs. 7-fold) [41,42]. More studies are required to evaluate the antigen processing and induction of antigen-specific T and B cell responses in the presence of AddaVax and AddaS03. Nevertheless, our results were in line with a recent study, which compared α-tocopherol-containing squalene o/w emulsion adjuvant (A-910823) with AddaVax to enhance recombinant SARS-CoV-2 spike protein-induced immune responses and found both adjuvants similarly enhanced spike protein-induced IgG and neutralizing antibody responses in murine models [40]. The more potent adjuvant effects of AS03 observed in clinical studies might be related to the different vaccines explored (avian influenza H5N1, H7N9, and H5N8 vaccines) or simply reflect the differential responses of humans and mice to squalene emulsion adjuvants [13,14,15].

## 5. Conclusions 

Our study indicates how subtle change of adjuvant formulation might affect their in vivo behavior, in particular when an immunostimulant (here α-tocopherol) is incorporated in the formulation. Our studies found α-tocopherol might stimulate MyD88-dependent pathways to enhance DC maturation and MyD88-independent pathways to mediate its other adjuvant effects. More work is needed to identify MyD88-dependent and -independent pathways to aid in a better understanding of AddaS03’s adjuvant effects. Overall, our work contributes to the understanding of the similarities and differences of the two squalene emulsion-based vaccine adjuvants in the potentiation of DC function and adaptive immunity, although it remains to be explored how the results obtained with AddaVax and AddaS03 can be translated to MF59 and AS03 adjuvants.

## Figures and Tables

**Table 1:** Comparison and droplet size of AddaVax and AddaS03 as well as their corresponding commercial products.

	AddaVax	MF59^®^	AddaS03	AS03^®^
Squalene	5%	4.3%	5%	4.3%
Span 85	0.5%	0.5%	-	-
Tween 80	0.5%	0.5%	1.8%	1.9%
α-tocopherol	-	-	5%	4.7%
Buffer	Citrate	Citrate	Phosphate	Phosphate
Size	~160 nm	~160 nm	~160 nm	~160 nm

## Data Availability

The data presented in this study are available on request from the corresponding author.

## Supplementary Materials

The following supporting information can be downloaded at: https://www.mdpi.com/article/10.3390/vaccines12050531/s1, Figure S1: AddaVax increases muscle DC levels; Figure S2: AddaS03 increases muscle CD80^hi^ DCs; Figure S3: AddaVax and AddaS03 slightly reduce CD40 and CD86 expression on muscle DCs; Figure S4: AddaS03 increases antigen uptake in popliteal LNs; Figure S5: AddaVax and AddaS03 fail to increase co-stimulatory molecule levels on migDC in popliteal LNs; Figure S6: AddaVax and AddaS03 fail to increase co-stimulatory molecule levels on cDC, migDC, or pDC in inguinal LNs; Figure S7: AddaVax and AddaS03 fail to increase MHC II levels on migDC or pDC.
