# Correction: Nakkala et al. Differential Regulation of DC Function, Adaptive Immunity, and MyD88 Dependence by Two Squalene Emulsion-Based Vaccine Adjuvants. *Vaccines* 2024, *12*, 531

**DOI:** 10.3390/vaccines12121388

**Published:** 2024-12-10

**Authors:** Jayachandra Reddy Nakkala, Yibo Li, Labone Akter, Xinliang Kang, Xinyuan Chen

**Affiliations:** Biomedical & Pharmaceutical Sciences, College of Pharmacy, University of Rhode Island, 7 Greenhouse Road, Avedisian Hall, Room 480, Kingston, RI 02881, USA; jayachandrareddy.nak@uri.edu (J.R.N.); yibo_li@uri.edu (Y.L.); labone_akter@uri.edu (L.A.); xinliang_kang@uri.edu (X.K.)

The authors would like to make the corrections described below to the published paper [1].

In the original manuscript, “AS03-like” has been replaced with “AddaS03” throughout the text (the Title, Abstract, Introduction, Method, Discussion, and Conclusion sections) to avoid confusion with the commercially approved AS03 adjuvant.

The title of the paper has been corrected to the following:

“Differential Regulation of DC Function, Adaptive Immunity, and MyD88 Dependence by Two Squalene Emulsion-Based Vaccine Adjuvants”

In the Abstract, the sentence “…, this study compared MF59 and AS03-like adjuvants (AddaVax and AddaS03, respectively) to enhance antigen uptake, DC maturation, ovalbumin (OVA) and seasonal influenza vaccine-induced immune responses” has been corrected to “…, this study compared AddaVax and AddaS03 with similar compositions to MF59 and AS03 adjuvants to enhance antigen uptake, DC maturation, ovalbumin (OVA), and seasonal influenza vaccine-induced immune responses”.

In the last paragraph of the Introduction, the last sentence should be as follows:

“Due to the crucial roles of dendritic cells (DCs) in bridging innate and adaptive immunity [23] and the unavailability of MF59 and AS03 adjuvants, this study explored the adjuvant effects of AddaVax and AddaS03 with similar compositions to MF59 and AS03 adjuvants to enhance the antigen uptake, DC maturation, vaccine-induced adaptive immunity, and MyD88 dependence of the diverse adjuvant effects.”

In a paragraph of Section 2.2, “Adjuvants”, the first sentence should be as follows:

“AddaVax (vac-adx-10) and AddaS03 (vac-as03-10) (both manufactured by Invivogen) were used in our studies due to the unavailability of MF59 and AS03 adjuvants.”

In the first paragraph of the Discussion, the first two sentences should be as follows: “Side-by-side comparison of AddaVax and AddaS03 adjuvants reveals their similarities and differences in potentiation of DC function and adaptive immunity. Our major findings are that the AddaVax adjuvant more significantly enhanced local antigen uptake, while the AddaS03 adjuvant more significantly enhanced antigen uptake in draining LNs; …”

In the third paragraph of the Discussion, “AS03-like” in the parentheses has been changed to “AddaS03”.

In the fourth paragraph of the Discussion, “AS03-like” has been removed in the fourth sentence.

In the last paragraph of the Discussion, the sentence “More studies are required to evaluate antigen processing and induction of antigen-specific T and B cell responses in the presence of the two adjuvants” has been changed to “More studies are required to evaluate the antigen processing and induction of antigen-specific T and B cell responses in the presence of AddaVax and AddaS03”. The last sentence has been changed to “The more potent adjuvant effects of AS03 observed in clinical studies might be related to the different vaccines explored (avian influenza H5N1, H7N9, and H5N8 vaccines) or simply reflect the differential responses of humans and mice to squalene emulsion adjuvants [13–15].”

In the Conclusions, the last two sentences should be as follows: “More work is needed to identify MyD88-dependent and -independent pathways to aid in a better understanding of AddaS03’s adjuvant effects. Overall, our work contributes to the understanding of the similarities and differences of the two squalene emulsion-based vaccine adjuvants in the potentiation of DC function and adaptive immunity, although it remains to be explored how the results obtained with AddaVax and AddaS03 can be translated to MF59 and AS03 adjuvants”.

The authors state that the scientific conclusions are unaffected. These corrections were approved by the Academic Editor. The original publication has also been updated.
