# Supplementary material for: Differential Regulation of DC Function, Adaptive Immunity, and MyD88 Dependence by Two Squalene Emulsion-Based Vaccine Adjuvants
Source: Vaccines (Basel). 2024 May 13;12(5):531. doi: 10.3390/vaccines12050531 (PMC11125884; doi:10.3390/vaccines12050531)
Supplement: Supplementary file 1 [file vaccines-12-00531-s001.zip › vaccines-2969567-supplementary.pdf]

Supplementary Information

Differential regulation of DC function, adaptive immunity, and MyD88 dependence by MF59 and AS03-like adjuvants

Nakkala et al.

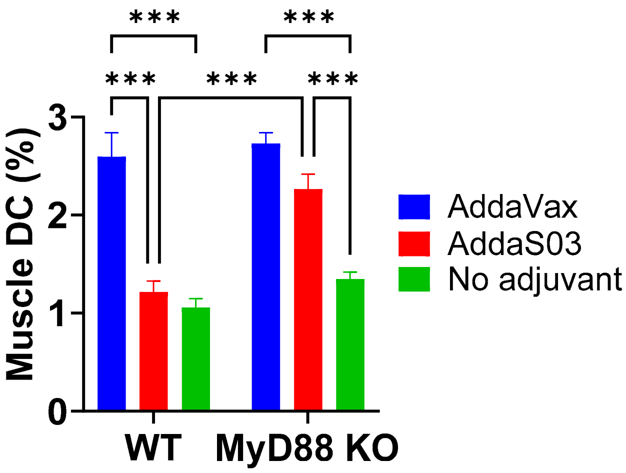

Fig.S1 AddaVax increases muscle DC levels

*Percentages of muscle CD11c^{+} MHC II^{+} cells in Fig.1 were compared between groups. Two-way ANOVA with Tukey's multiple comparison test was used to compare difference between groups. n=6. ***, $p<0.001$.*

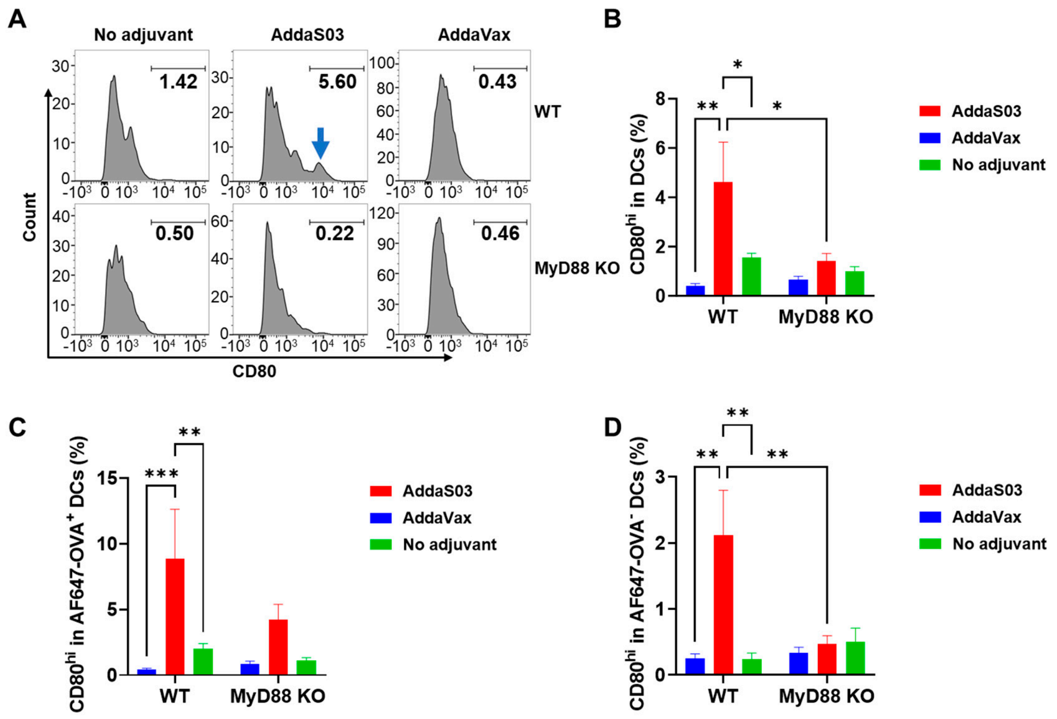

Fig.S2 AddaS03 increases muscle CD80^{hi} DCs

Muscle cells were analyzed for percentage of CD80^{hi} DCs. Live cells were first gated based on FSC and SSC. Muscle DCs were then gated based on CD11c and MHC II expression. CD80^{hi} cells were then gated. ***A****. Representative histograms about CD80^{hi} cells in muscle DCs in WT (top) and MyD88 KO mice (bottom). Arrow: Induced peak of CD80 expression.* ***B****. Percentage of CD80^{hi} cells in muscle DCs.* ***C-D****. DCs were further gated based on AF647-OVA. Percentage of CD80^{hi} DCs was further analyzed in AF647-OVA^{+}(****C****) and AF647-OVA^{-} DCs (****D****). Two-way ANOVA with Tukey's multiple comparison test was used to compare differences between groups. n=6. *, $p<0.05$; **, $p<0.01$; ***, $p<0.001$.*

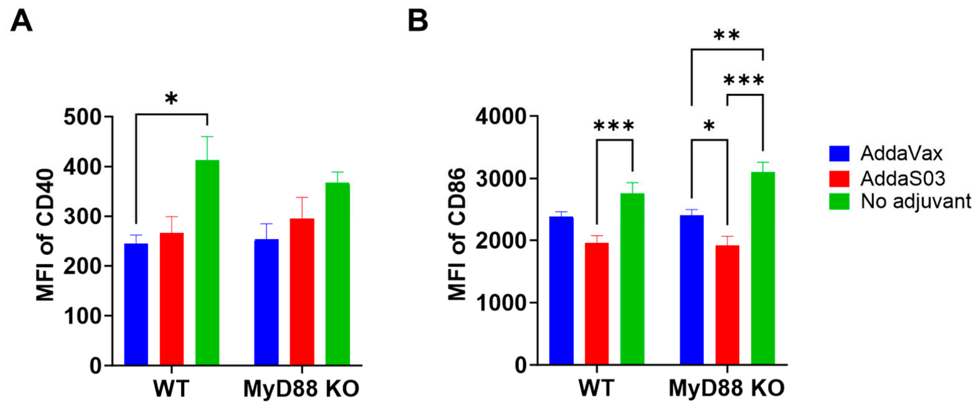

Fig.S3 AddaVax and AddaS03 slightly reduce CD40 and CD86 expression on muscle DCs

*MFI of CD40 (**A**) and CD86 (**B**) on muscle $CD11c^+$ MHC II^+ cells from mice in Fig.2 was compared between groups. Two-way ANOVA with Tukey's multiple comparison test was used to compare differences between groups. n=6. *, p<0.05; **, p<0.01; ***, p<0.001.*

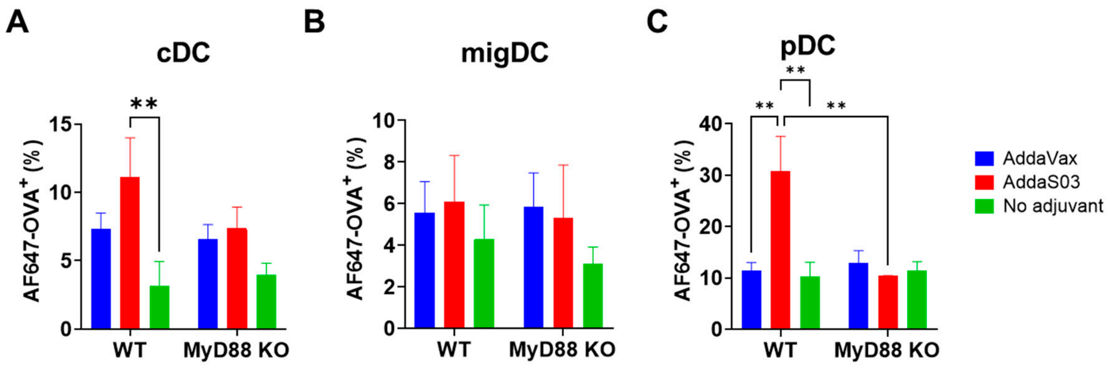

Fig.S4 AddaS03 increases antigen uptake in popliteal LNs

Popliteal LNs were collected 15 h after intramuscular injection of AF647-OVA alone or in the presence of AddaVax or AddaS03. Single-cell suspensions were prepared followed by immunostaining and flow cytometry analysis as in Fig.3. Percentage of AF647-OVA^{+} *cells in cDC (A), migDC (B), and pDC (C) were compared between groups. Two-way ANOVA with Tukey's multiple comparison test was used to compare difference between groups. n=6. **, p<0.01.*

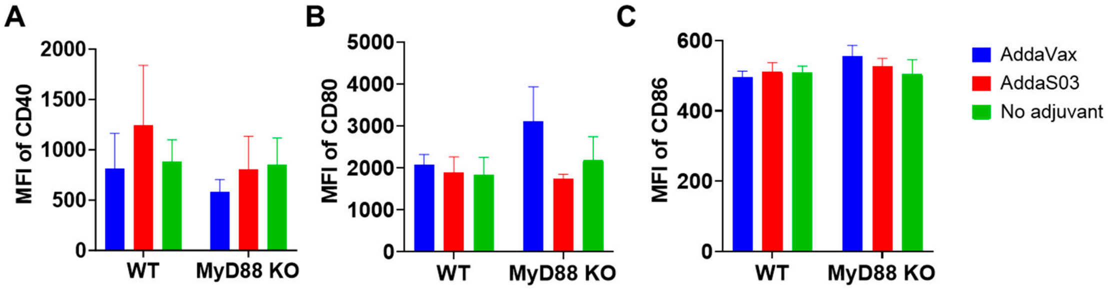

Fig.S5 AddaVax and AddaS03 fail to increase co-stimulatory molecule levels on migDC in popliteal LNs

Lymphocytes were stained and analyzed by flow cytometry as in Fig.4. MFI of CD40 (A), CD80 (B), and CD86 (C) in migDC in popliteal LNs were shown. Two-way ANOVA with Tukey's multiple comparison test was used to compare difference between groups. n=6.

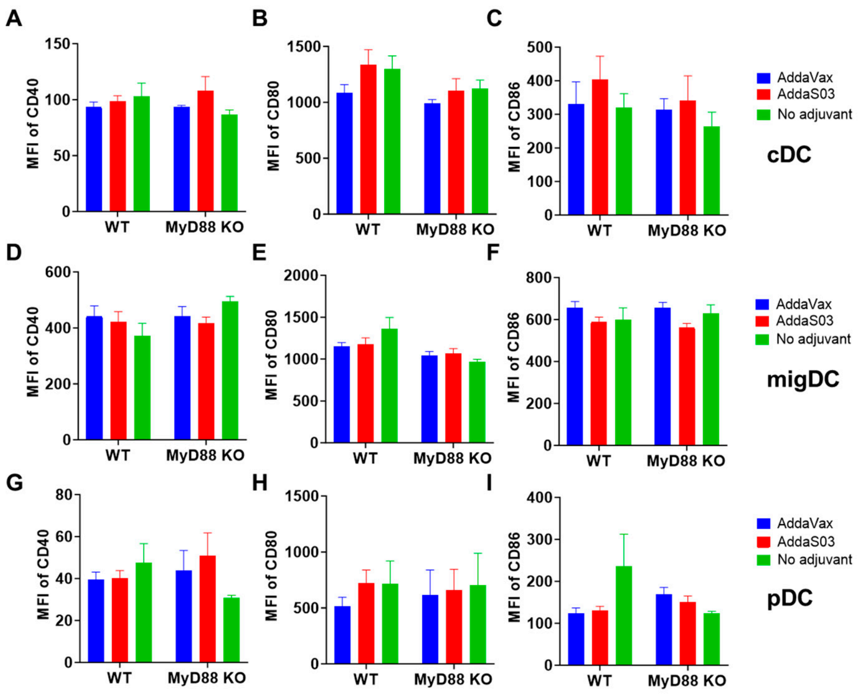

Fig.S6 AddaVax and AddaS03 fail to increase co-stimulatory molecule levels on cDC, migDC, or pDC in inguinal LNs

Lymphocytes were stained and analyzed by flow cytometry as in Fig.4. MFI of CD40 (A, D, G), CD80 (B, E, H), and CD86 (C, F, I) in cDC (top), migDC (middle), and pDC (bottom) in inguinal LNs were shown.

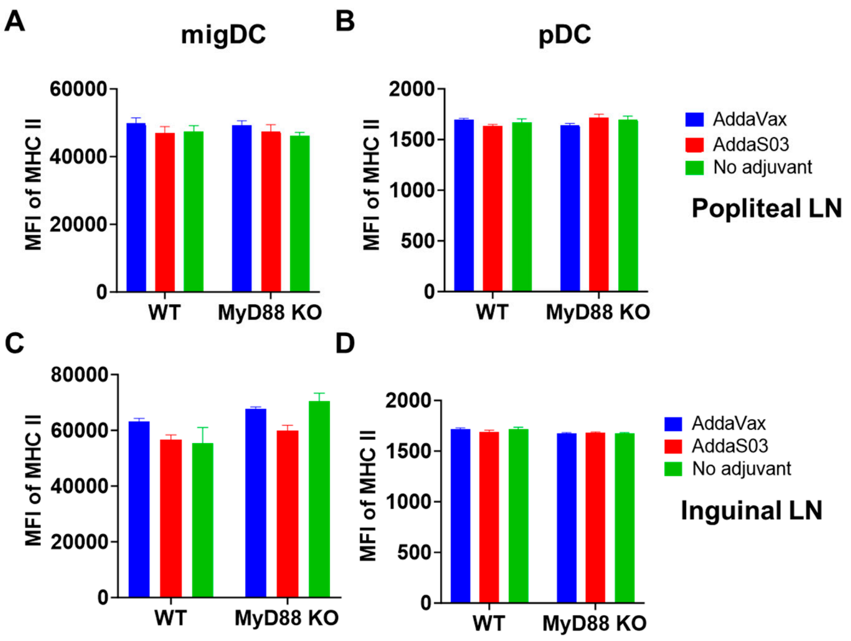

Fig.S7 AddaVax and AddaS03 fail to increase MHC II levels on migDC or pDC

Lymphocytes were stained and analyzed by flow cytometry as in Fig.5. MFI of MHC II in migDC (A, C) and pDC (B, D) in popliteal (top) and inguinal LNs (bottom) were shown.
